# Efficacy and Skin Acceptability of a Cosmetic Cream for Nasolabial Dryness and Irritation

**DOI:** 10.1111/jocd.70107

**Published:** 2025-04-09

**Authors:** Constance Prime, William Hooper, Yosra Ghrib Ben Sassi, Nejib Doss, Oliver Chen

**Affiliations:** ^1^ Sofibel‐Church & Dwight Levallois‐Perret France; ^2^ Church & Dwight Princeton New Jersey USA; ^3^ Eurofins, Dermscan Ariana Tunisie; ^4^ Eurofins, Dermscan Tunis Tunisie

**Keywords:** facial skin, nasal irritation, repair, rhinorrhea, skin barrier, skincare, topical

## Abstract

**Introduction:**

Rhinorrhea is a widespread condition related to common upper respiratory insults such as influenza and rhinitis, often accompanied by frequent nose cleansing resulting in skin irritation. This study evaluated the cutaneous acceptability and nourishing, soothing, protecting, and moisturizing effects of Sterimar Soothing Nose Cream on the skin around the nose in healthy adults.

**Methods:**

We conducted an exploratory single‐arm clinical trial in 33 adults (mean age 46 years) who blow their nose often (> 5 times/day) due to a cold or allergies and have sensitive, irritated/dry nose. Participants applied the cream topically on their noses twice daily for 14 days. Cutaneous acceptability and efficacy measures were assessed by participants and by the study dermatologist before, during, and after 14 days. Skin imaging analysis via the Visia camera system and skin hydration via Corneometer were also assessed. Participants also completed a questionnaire about overall product performance.

**Results:**

The dermatologist noted reduced (*p* < 0.001) redness, swelling, dryness, roughness, and itchiness, with improved (*p* < 0.001) hydration, softness, and flexibility after 7 and 14 days. Participants reported less (*p* < 0.05) dryness, redness, and discomfort, with 97% rating the cream's soothing effect as moderate to strong. The cream also increased (*p* < 0.0001) skin hydration by 14%, measured by Corneometer 30 min postapplication. No worsening of symptoms or adverse effects was reported. All participants found the cream pleasant, with most intending to purchase and continue use.

**Conclusions:**

The results indicate that Sterimar soothing nose cream is effective in nourishing, soothing, protecting, and moisturizing the nasolabial zone, and is well tolerated.

## 1 Introduction

Rhinorrhea occurs when the mucous membranes of the nose are either inflamed or irritated. This can directly or indirectly affect mucus‐producing cells called goblet cells [1, 2]. Inflammation can occur in response to an infection or allergies such as the common cold or allergic rhinitis. When the nasal passages are affected, it can overstimulate goblet cells, causing them to produce more mucus than normal. Overproduction of mucus by the goblet cells also occurs when they are exposed to irritants such as noxious household fumes, industrial chemicals and fumes, and aerosolized capsaicin and other hot spices to help clear the irritants from the nasal passage. Rhinorrhea may also occur due to old age (geriatric rhinitis) and for unspecified reasons (vasomotor rhinitis) [3]. Rhinorrhea is a widespread and commonplace condition. Billions of people worldwide are affected by cold/flu and allergic rhinitis, and an unaccounted number of people are exposed to irritants daily [4, 5].

Rhinorrhea is often accompanied by frequent nose cleansing with tissue or cloth, resulting in mechanically induced dermatitis of the area around the nose. Repetitive rubbing of the skin around the nose and mouth can strip away the protective hydrolipidic layer and compromise the barrier function of the upper stratum corneum. This damage to the skin barrier immediately triggers the release of pro‐inflammatory cytokines, chemokines, and growth factors, stimulating the migration and proliferation of immune cells [6]. Activation of inflammatory signaling pathways results in further production of cytokines, chemokines, and eicosanoids, such as prostaglandins and leukotrienes, intensifying the inflammatory response [6]. Moreover, various mediators, including vascular endothelial growth factor, nitric oxide, and histamine, are produced, causing vasodilation, swelling, mast cell degranulation, pain, and itching, exacerbating inflammation and skin sensitivity [6]. Concurrently, this process can disrupt the local microbiota, leading to an imbalance in the skin's microbial community, which in turn disrupts the immune system and fosters the growth of harmful microorganisms, increasing the risk of secondary infections [7].

Nasolabial skin damage can be managed using various approaches. Less frequent nose cleansing with tissue or cloth or by using gentler materials would cause less abrasion of the nasolabial zone [8, 9]. Another approach is to treat nasolabial skin dryness and irritation through the application of cosmetic products (creams or balms) that claim to protect, hydrate, and restore skin. Ideally, the product would facilitate the repair of the damaged skin and mitigate or limit further damage, such as by repairing the skin barrier, adding and/or maintaining skin moisture, and decreasing inflammation. Several of these functions can be achieved by botanicals [10]. Indeed, Church and Dwight has developed a nose cream (Sterimar soothing nose cream) consisting of a proprietary blend of several functional botanicals, such as sweet almond ( *Prunus amygdalus* var. dulcis) oil, *aloe vera* ( *Aloe barbadensis* Miller), chamomile ( *Chamomilla recutita* ), and *Spirulina platensis*. The Sterimar Soothing Nose Cream was designed to soothe irritated skin, moisturize, and protect the skin around the nose while reducing redness. Despite the frequent use of these functional botanicals in skincare formulations, the effect of the unique combination of sweet almond oil, aloe, chamomile, and spirulina on nasolabial skin damage is unknown. Additionally, it is essential to clinically evaluate topical cosmetic products for the risk of adverse reactions. Thus, the present study aimed to evaluate the efficacy and the cutaneous acceptability and nourishing, soothing, protecting, and moisturizing effect of a nose cream (Sterimar Soothing Nose Cream) on the skin around the nose of healthy men or women who blow their nose often (> 5 times/day) due to a cold or allergies and have a sensitive, irritated/dry nose.

## 2 Materials and Methods

### 2.1 Study Design

This study was a one‐arm, open‐label clinical trial conducted in 33 healthy adults suffering from a sensitive, irritated/dry nose. The study was conducted following the Helsinki Declaration (1964) and its successive updates and the guidance on Good Clinical Practice CPMP/ICH/135/95 (R2). Signed informed consent and authorization for the use of protected health information were provided by the participants before implementing any protocol‐specific procedures. The study was conducted between January 25, 2022 and February 24, 2022 at a single clinical research site (Eurofins Dermscan; Tunis, Tunisia).

### 2.2 Study Participants

Participants were healthy men or women, 18–65 years old, who blow their nose often (> 5 times/day) due to a cold or allergies and have sensitive, irritated/dry nose with at least a mild score for at least two of the irritation symptoms: redness, dryness, itching, roughness, desquamation, and/or edema. Exclusion criteria included pregnant, nursing, or planning to get pregnant, presenting a cutaneous pathology on the study zone (e.g., eczema), having surgery under general anesthesia within the previous month, being exposed to sunlight or ultraviolet rays (including artificial) > 2 h/day in the last month, planning to use another topical treatment on the nose area during the study, enrollment in another clinical trial during the study period, and using topical or systemic treatments during the previous weeks that were liable to interfere with the assessment of the cutaneous acceptability/efficacy of the study test product. Participants were allowed to use antihistamines or medications to treat common cold symptoms and were instructed to maintain their usual makeup and hygiene routines. The use of the medications was inquired about during the study visits. Before each visit, participants were instructed to forgo their habitual facial skin care routine except for their habitual morning wash with their usual cleaning products.

### 2.3 Study Product

The nasal cream was an emulsion formulated with aqua, cetyl alcohol, stearyl alcohol, glyceryl stearate SE, isopropyl palmitate, ceteareth‐20, phenoxyethanol, glycerin, allantoin, *prunus amygdalus dulcis* oil, tartaric acid, sodium citrate, citric acid, *spirulina platensis* extract, maltodextrin, sodium benzoate, *aloe barbadensis* leaf powder, and *chamomilla recutita* (matricaria) flower extract (Stérimar, Sofibel Laboratoires Fumouze, Levallois‐Perret, France). Participants were instructed to apply the cream in a fine layer to the irritated area or the area that needed to be soothed at least twice a day and massage it in gently. They were asked to avoid applying the cream inside the nasal cavity and around the eyes. The first application was done by a trained research assistant at the baseline visit (Visit 1, Day 0), and the last application was completed the day before Visit 3 (i.e., the last application was on Day 13). The study cream was weighed before use (Day 0) and at the end of the study (Day 14) to confirm the use of the nasal cream by the participants. Compliance was determined based on the number of applications recorded in the daily log as well as the weight of the product at the end of the study. Noncompliance was defined as < 80% of scheduled use (per daily log record or by weight).

### 2.4 Measurements

#### 2.4.1 Skin Examination by Dermatologist

**FIGURE 1:**
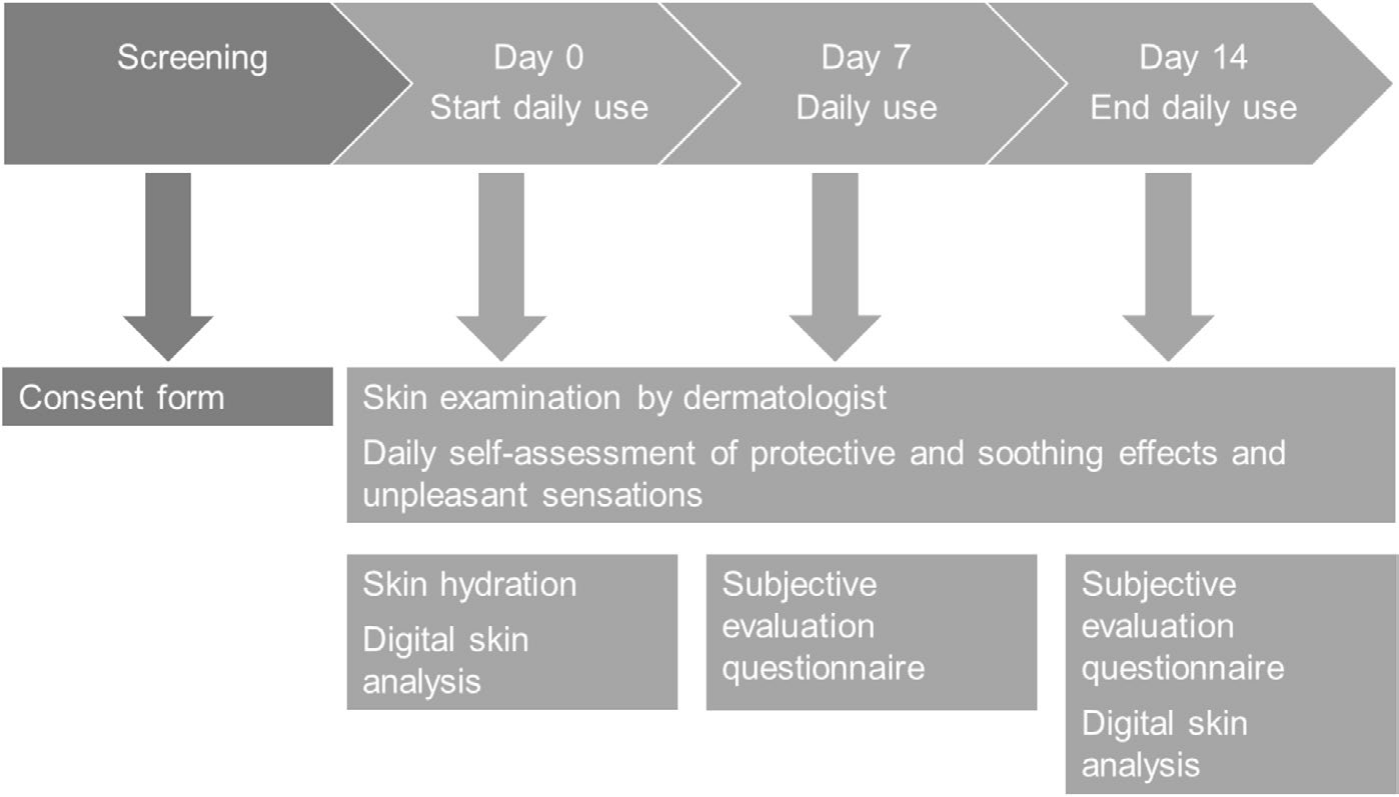
Flow chart of study procedures.

On Day 0 (before product application) and on Days 7 and 14, the study dermatologist, Dr. Nejib Doss, at Eurofins Dermscan performed a visual skin examination on the area of product application for the presence of irritation signs (i.e., redness, edema, dryness, desquamation, and roughness) (Figure 1). The results on Day 0 were used to confirm the eligibility of study participants. The severity of the signs was scored using the 5‐point scale: 0 = none; 1 = very mild; 2 = mild; 3 = moderate; 4 = severe. Additionally, the dermatologist also graded visual hydration/dryness, tactile suppleness, and tactile softness on an 11‐point scale on Days 0, 7, and 14, whereby a higher score is desirable. During the same study days, the study dermatologist inquired of participants about the presence of irritation signs (i.e., tightness, stinging, itching, warm burning sensation, redness, edema, dryness, desquamation, and roughness).

#### 2.4.2 Self‐Assessment of Protective and Soothing Effects and Unpleasant Sensations

From Day 0 through Day 14, participants completed a daily self‐assessment of the protecting effects by evaluating the presence and severity of dryness, redness, and signs of discomfort. The severity of the signs was scored using a 5‐point scale of either 0 = none; 1 = very mild; 2 = mild; 3 = moderate; 4 = severe or 0 = comfortable skin; 1 = very mild uncomfortable, 2 = mild uncomfortable, 3 = moderate uncomfortable, 4 = uncomfortable skin. Participants were also asked to self‐assess daily the presence of a soothing effect of the cream (yes/no) and, if present, to rate the intensity of the effect (1 = very mild; 2 = mild, 3 = moderate, 4 = strong) immediately after the first product application on Day 0 and on Days 7 and 14.

#### 2.4.3 Subjective Evaluation Questionnaire

On Days 7 and 14, participants completed a questionnaire on product attributes (e.g., the texture of the product is pleasant, the product is easy to apply) and overall product efficacy (e.g., the product reduces redness, the skin is moisturized). All questions required the selection of one of four options: agree; somewhat agree; somewhat disagree; and disagree. Additionally, on Day 14 only, participants were asked to evaluate an overall assessment of the product (very pleasant, pleasant, unpleasant, very unpleasant) and future use of the product (i.e., would you like to continue to use the product? Yes/No; would you buy the product (regardless of its price)?).

#### 2.4.4 Skin Hydration

On Day 0, skin hydration was measured by a trained Eurofins Dermscan technician using a Corneometer CM 825 (Courage+Khazaka electronic GmbH, Köln, Germany). The measurement was performed to evaluate the acute hydration effect of the study cream. Measurements were performed before and after the application of the study cream on the participants' forearm (4‐cm^2^ surface). The control was an area of the forearm where no cream was applied. The study cream was left on the skin for 30 min and then cleaned using a damp cloth. After air drying the area, skin hydration was measured.

#### 2.4.5 Digital Skin Analysis

On Days 0 and 14, facial capture was conducted using the Visia CAS imaging system (Canfield Scientific, Parsippany, NJ, USA) by a trained Eurofins Dermscan technician in ambient conditions. The control of the facial repositioning took place directly on the data‐processing screen using an overlay visualization of the images at each time of acquisition. On Day 0, facial imaging was performed before applying the study cream. Facial imaging was again performed on Day 14. Results from before and after 14 days of application were visually compared.

### 2.5 Statistical Analysis

Statistical analysis was performed using SAS (Version 9.4), and figures were generated with Minitab (Version 21.2). Primary outcomes included the cutaneous acceptability and efficacy of the cream. Acceptability was assessed through dermatologist evaluations of redness, edema, dryness, desquamation, and roughness (baseline, Day 7, and Day 14) and participant‐reported sensations (e.g., tightness, stinging, itching). Efficacy included nourishing, soothing, protecting, and moisturizing effects, assessed via dermatologist evaluations, participant self‐assessments, and hydration measurements before and after cream application. Additional outcomes included subjective ratings of attributes, efficacy, and likelihood of future use (Days 7/14).

Efficacy was analyzed using the Wilcoxon signed‐rank test for changes from baseline (*p* ≤ 0.05). Daily diary scores of dryness, redness, and discomfort were analyzed via ANOVA with Dunnett's method for multiple comparisons. Descriptive statistics (mean, SEM, frequency) were calculated for self‐assessments, with Fisher's exact test used for proportion comparisons (*p* ≤ 0.05). Responses on product attributes, efficacy, and future use were analyzed with 95% confidence intervals using the Wilson method.

## 3 Results

### 3.1 Panel Characteristics

Thirty‐three participants were enrolled and completed the study. Their characteristics are summarized in Table 1.

**TABLE 1:** Participant demographic and skin characteristics.

Characteristics		Nasal cream (*n* = 33)
Age, (years)	Mean (minimum, maximum)	46 (24, 64)
Sex, *n* (%)	Male	5 (15.2)
	Female	28 (84.8)
Nose skin type, *n* (%)	Normal	55
	Dry	33
	Greasy	12
Skin phototype, *n* (%)	II	12
	III	67
	IV	21

Abbreviation: *n*, sample size.

### 3.2 Cutaneous Acceptability

The changes from baseline (Day 0) of the visual assessments by the study dermatologist on Day 7 and Day 14 are presented in Table 2. Scoring for redness, edema, dryness, roughness, and itching significantly decreased (*p* < 0.001) after applying the cream on Day 7 and Day 14. For desquamation, tightness, and stinging, no participant had any nonzero scores on either Day 7 or Day 14, and fewer than 11 of 33 participants were scored as having any changes from baseline, which was insufficient data for statistical analysis. During their self‐assessments, all 33 participants reported that none of these acceptability signs worsened by Day 7 or Day 14 compared to Day 0.

**TABLE 2:** Change from baseline (Day 0) of clinical signs assessed by dermatologists on Day 7 and Day 14.

		Mean score (±SEM)	Mean change from baseline (±SEM)	*p* ^a^
Redness	Day 0	2.1 ± 0.0		
	Day 7	0.9 ± 0.1	−1.2 ± 0.1	< 0.001
	Day 14	0.4 ± 0.1	−1.7 ± 0.1	< 0.001
Edema	Day 0	0.5 ± 0.1		
	Day 7	0.1 ± 0.1	−0.5 ± 0.1	< 0.001
	Day 14	0.0 ± 0.0	−0.5 ± 0.1	< 0.001
Dryness	Day 0	1.7 ± 0.1		
	Day 7	0.1 ± 0.1	−1.5 ± 0.1	< 0.001
	Day 14	0.1 ± 0.0	−1.6 ± 0.1	< 0.001
Desquamation	Day 0	0.3 ± 0.1		
	Day 7	0.0 ± 0.0	−0.3 ± 0.1	NA^b^
	Day 14	0.0 ± 0.0	−0.3 ± 0.1	NA
Roughness	Day 0	1.0 ± 0.0		
	Day 7	0.0 ± 0.0	−1.0 ± 0.0	< 0.001
	Day 14	0.0 ± 0.0	−1.0 ± 0.0	< 0.001
Tightness	Day 0	0.2 ± 0.1		
	Day 7	0.0 ± 0.0	−0.2 ± 0.1	NA
	Day 14	0.0 ± 0.0	−0.2 ± 0.1	NA
Stinging	Day 0	0.3 ± 0.1		
	Day 7	0.0 ± 0.0	−0.3 ± 0.1	NA
	Day 14	0.0 ± 0.0	−0.3 ± 0.1	NA
Itching	Day 0	1.0 ± 0.2		
	Day 7	0.0 ± 0.0	−1.0 ± 0.2	< 0.001
	Day 14	0.0 ± 0.0	−1.0 ± 0.2	< 0.001

Abbreviations: NA, not applicable; SEM, standard error of the mean.

^a^ Wilcoxon signed‐rank test for change from baseline.

^b^ No subject had any nonzero scores at either Day 7 or Day 14, and fewer than 11 of the 33 subjects experienced any changes from baseline, which was insufficient data for statistical analysis.

### 3.3 Nourishing, Hydrating, Soothing, and Protecting Efficacy

By Day 7 and Day 14, the cream induced a significant increase (*p* < 0.001) in all nourishing‐related measurements assessed by the study dermatologist (i.e., hydration/dryness, softness, and suppleness scores; Table 3). Skin hydration scores increased by 66% and 96% on Day 7 and Day 14, respectively, compared to the baseline. Skin softness scores increased by 36% (Day 7) and 69% (Day 14) and skin suppleness was improved by 38% (Day 7) and 71% (Day 14).

**TABLE 3:** Scores for hydration, softness, and suppleness assessed by dermatologists based on visual and tactile assessment on Days 0 (baseline), 7 and 14.

		Mean (±SEM)	Mean change from baseline (±SEM)	*p* ^a^
Hydration	Day 0	4.2 ± 0.1		
	Day 7	7.0 ± 0.0	2.8 ± 0.2	< 0.001
	Day 14	8.2 ± 0.1	4.0 ± 0.2	< 0.001
Softness	Day 0	4.2 ± 0.2		
	Day 7	5.7 ± 0.1	1.5 ± 0.2	< 0.001
	Day 14	7.1 ± 0.1	2.9 ± 0.2	< 0.001
Suppleness	Day 0	4.1 ± 0.2		
	Day 7	5.7 ± 0.1	1.5 ± 0.2	< 0.001
	Day 14	7.0 ± 0.1	2.9 ± 0.2	< 0.001

Abbreviation: SEM, standard error of the mean.

^a^ Wilcoxon signed‐rank test for change from baseline.

**FIGURE 2:**
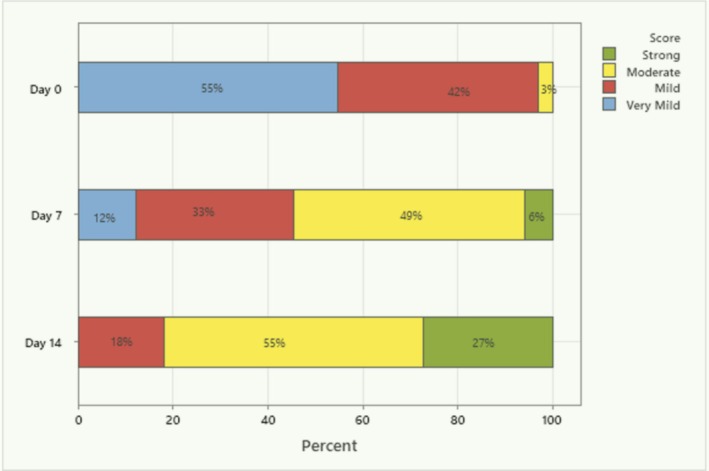
Percentage of participants that reported the soothing effect as strong, moderate, mild, or very mild immediately post the first application (Day 0) and after 7 days (Day 7), and 14 days (Day 14) of application.

Overall, there was a statistically significant shift in the perception of the soothing effect from Day 0 to Day 7 and Day 7 to Day 14 (Figure 2). On Day 0, only one subject (3%) experienced either a “moderate” or “strong” effect. This increased to 18 subjects (55%) on Day 7 and to 27 subjects (82%) on Day 14, with the Day 14 total statistically significantly greater than 50% (*p* < 0.05). The increase in “moderate” to “strong” effect from Day 0 to Day 7 was statistically significant (*p* < 0.05), as was the increase from Day 7 to Day 14 (*p* < 0.05).

**FIGURE 3:**
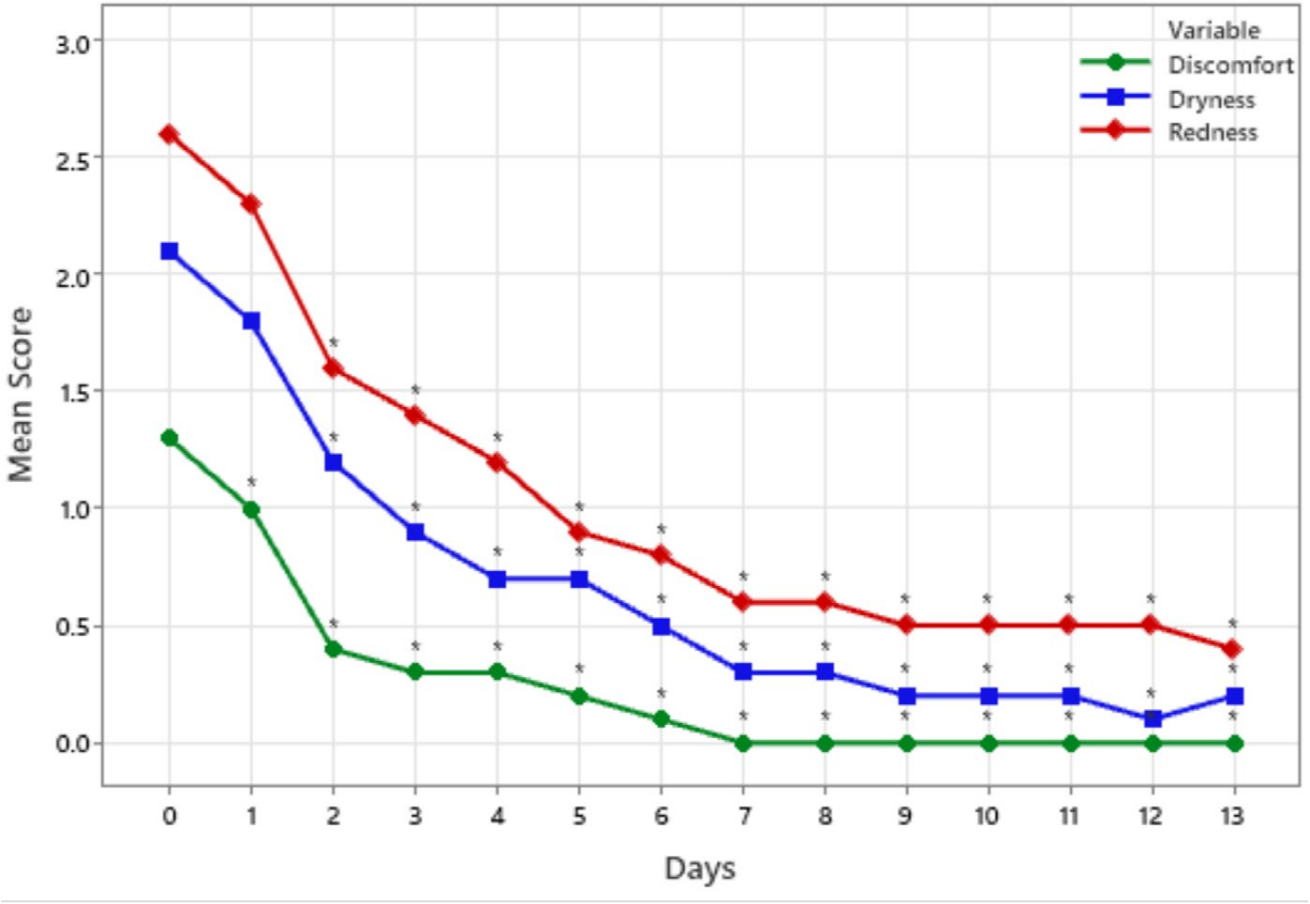
Mean scores of the self‐assessment of dryness, redness, and discomfort (0 = none; 1 = very mild; 2 = mild; 3 = moderate; 4 = severe). *p < 0.05.

Mean scores of the daily self‐assessed protecting effects (i.e., reduction in dryness, redness, and discomfort) are presented in Figure 3. Overall, the protective effects of the cream increased with use. Discomfort scores from Day 1 to Day 13 were significantly lower than those from Day 0 (*p* < 0.05). Redness and dryness scores from Day 2 to Day 13 were significantly lower than those from Day 0 (*p* < 0.05).

### 3.4 Moisturizing Efficacy

The application of the cream on the forearm for 30 min increased the hydration rate as measured by a Corneometer. The hydration rate of the clean, control skin surface was unchanged (*p* = 0.365) on the skin surface. Following the application of the cream, the hydration rate significantly increased (*p* < 0.0001) from 21 ± 25 to 24 ± 27 (arbitrary units) on the skin surface compared to before the application of the cream. This 14% increase in hydration rate was significantly different compared to the control (*p* < 0.0001).

### 3.5 Skin Imaging Using VISIA

**FIGURE 4:**
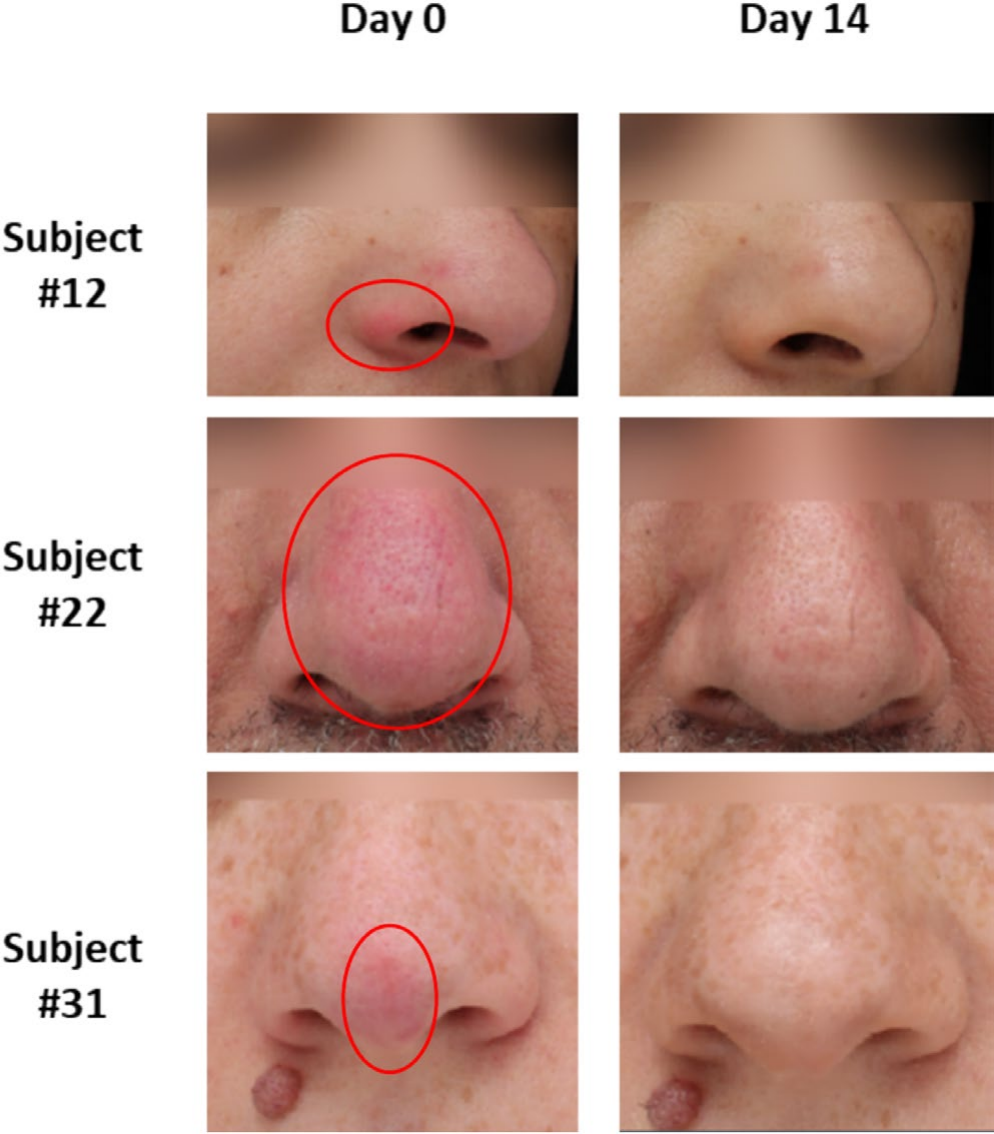
Change of the nose redness with a red circle in three representative participants before (Day 0) and after (Day 14) the use of the study nasal cream, illustrated by VISIA photographs.

The visual evolution of skin redness around the nose of three representative participants is illustrated in Figure 4. The redness of the skin around the nose was improved on Day 14, supporting the statistically significant improvements in skin redness reported by the dermatologist (Table 2) and study participants (Figure 3).

### 3.6 Subjective Evaluation of Attributes, Overall Efficacy, and Future Use

A majority of the participants either agreed or somewhat agreed with the beneficial sensorial attributes of the cream (Table 4). Additionally, most participants provided positive feedback related to efficacy, comfort, and overall acceptance of the cream (Table 5). When asked directly about their overall assessment of the cream, all 33 participants responded positively (45% pleasant and 55% very pleasant, combined confidence interval of 90% ‐ 100%), while 31 out of 33 (94%) wanted to purchase and continue using the cream.

**TABLE 4:** Percentage of participants who have positive responses (agree or somewhat agree) about the sensorial properties of the study product.

Statement	After 14 days of use	95% CI
The texture of the product is pleasant	100%	90%–100%
The skin is not shiny after application	100%	90%–100%
The texture of the product is not oily	100%	90%–100%
The scent of the product is pleasant	100%	90%–100%
The texture of the cream is nonsticky	100%	90%–100%
The texture of the cream is smooth	100%	90%–100%
The product is easy to apply	97%	85%–99%
The product penetrates quickly	97%	85%–99%

Abbreviation: CI, confidence interval.

**TABLE 5:** Percentage of participants with positive feedback related to efficacy, comfort, and overall acceptance of the study product.

	Statement	Day 7	Day 14
		Percentage (95% CI)	Percentage (95% CI)
1	The product provides an optimal feeling of comfort	85% (69%–93%)	94% (80%–98%)
2	The skin is moisturized	94% (80%–98%)	94% (80%–98%)
3	The product soothes the skin	88% (73%–95%)	94% (80%–98%)
4	The product reduces redness	100% (90%–100%)	100% (90%–100%)
5	The product prevents the appearance of skin reactions	88% (73%–95%)	91% (77%–97%)
6	The product reduces sensations of skin discomfort	91% (77%–97%)	88% (73%–95%)
7	The skin feels repaired	97% (85%–99%)	91% (77%–97%)
8	The skin is protected from drying out	NA^a^	91% (77%–97%)
9	The product does not make the skin shiny	91% (77%–97%)	100% (90%–100%)
10	A tube format would be more convenient for me	NA^a^	100% (90%–100%)
11	The product is suitable for me	NA^a^	97% (85%–99%)
12	I would continue to use this product	NA^a^	94% (80%–98%)
13	I would purchase the product (regardless of its price)	NA^a^	94% (80%–98%)
14	Overall assessment of the product (pleasant or very pleasant)	NA^a^	100% (90%–100%)

Abbreviations: CI, confidence interval; NA, not applicable.

^a^ Not applicable as these were not asked on Day 7.

## 4 Discussion

In this prospective, single‐arm, open‐label, single‐center, clinical trial, 33 adult male and female participants who blow their nose often (> 5 times/day) due to a cold or allergies, and have sensitive, irritated/dry nose were enrolled to evaluate the efficacy and cutaneous acceptability of a new nose cream. The study focused on assessing the cutaneous acceptability and nourishing, soothing, protecting, and moisturizing efficacy in the nasolabial zone of the cream under normal usage conditions. After 7 and 14 days, dermatologists noted significant reductions in redness, edema, dryness, roughness, and itching, with increased hydration, softness, and suppleness. Participants reported decreased dryness, redness, and discomfort, with 97% rating the cream's soothing effect as moderate to strong. The cream also significantly increased hydration, as measured by Corneometer. There were no adverse events or worsening of symptoms. All participants found the cream pleasant, and most would purchase and continue using it.

The nourishing, soothing, protecting, and moisturizing effects of the cream are attributed to the unique properties of its active ingredients. Sweet almond oil, for instance, has been demonstrated to reduce transepidermal water loss (TEWL), indicating its ability to semi‐occlude the skin surface, thus aiding in moisture retention [11, 12]. TEWL is a widely used measure of epidermal permeability barrier function whereby the decrease in TEWL represents a well‐functioning skin barrier, keeping the skin properly hydrated [13]. Moreover, the topical use of sweet almond oil reduced uremic pruritus in hemodialysis patients, likely through an anti‐inflammatory mechanism [14]. Because the inflammatory response exacerbated by frequent nose cleansing with tissue or cloth causes skin damage, the anti‐inflammatory property of sweet almond oil is expected to aid in skin repair [6]. Aloe is a tropical succulent plant widely utilized in treating various skin disorders [15]. Topical application of aloe extract enhances hydration and skin barrier function within 2 weeks, while oral consumption of aloe contributes to skin health by significantly improving skin elasticity, collagen levels, moisturization, and TEWL after a 12‐week regimen [16, 17]. Additionally, aloe exhibits anti‐inflammatory properties attributable to its sterols, which are expected to aid with skin repair [18]. Another ingredient found in the cream is chamomile, which is widely employed in treating skin and mucous membrane inflammations and skin damage, including diaper rash and cracked nipples, as well as various bacterial skin infections, oral cavity and gum issues, and respiratory tract ailments [19]. Comparative studies have suggested that chamomile may facilitate faster and more complete wound healing than corticosteroids [20]. Lastly, spirulina‐based formulations offer a multitude of benefits, including antioxidant, revitalizing, moisturizing, protective, cleansing, and shining actions for both hair and skin [21]. Studies conducted on rats and cell cultures have demonstrated significant wound healing properties of spirulina extracts, particularly in stimulating cell proliferation and migration [21]. Taken together, these functional botanicals possess properties that contribute to the observed nourishing, soothing, protecting, and moisturizing effects of the nasal cream, such as increasing moisture retention, decreasing inflammation, and stimulating cell proliferation and migration.

In this study, the patients reported no side effects. This result aligns with other evidence on the functional botanicals. The topical applications of sweet almond oil, aloe, and chamomile have been regarded as safe as assessed by the Cosmetic Ingredient Review Expert Panel [22, 23, 24]. It should be noted that several of the referenced skin irritation/sensitization studies (unpublished data presented to the committee) [22] noted occasional skin redness following the application of sweet almond oil, a side effect that was absent in our study. There are few reports about the safety of topical spirulina. In vitro toxicity studies of spirulina suggest no cytotoxicity or irritant potential, which were confirmed in the in vivo acceptability tests, which showed no alteration in skin barrier function and no report of irritation perception of signs of erythema [25]. The Dietary Supplements Information Expert Committee (DSI‐EC) of the United States Pharmacopeial Convention (USP) assigned a Class A safety rating for oral intake of *Spirulina platensis*, indicating that the available evidence does not indicate a serious health risk [26].

A strength of this study is the use of evaluations by a dermatologist as well as self‐reported outcomes by participants. Furthermore, these subjective evaluations were supported by objective measurements of hydration by Corneometer as well as skin imaging using VISIA. This study demonstrated excellent treatment compliance, with all participants successfully completing the trial. The total absence of adverse events and skin reactions during this period underscores the product's tolerability among individuals with sensitive nasolabial skin. This study is limited by subject demographics, the absence of a control formulation, and a relatively small number of subjects for product attribute assessments. The study did not include a placebo arm because the tested nasal cream contains vehicle ingredients with skin‐softening properties, including the emollients cetyl alcohol, stearyl alcohol, and isopropyl palmitate. Conducting a clinical study with a larger sample size and a longer duration will allow for a more in‐depth analysis of product attributes.

In conclusion, the results indicate that Sterimar soothing nose cream is effective in nourishing, soothing, protecting, and moisturizing the nasolabial zone and is well tolerated by adults who blow their nose often (> 5 times/day) and have sensitive, irritated/dry nose. The cream also has high consumer acceptability, with a majority of participants indicating that they would purchase the cream.

## Author Contributions

C.P. contributed to the product design conceptualization. O.C. contributed to study design, study management, and manuscript preparation. W.H. contributed to data analysis. Y.G.B.S. and N.D. contributed to the study conduct. All authors have read and accepted the published version of the manuscript.

## Ethics Statement

The study was conducted following the Helsinki Declaration (1964) and its successive updates, and the guidance on Good Clinical Practice CPMP/ICH/135/95 (R2). Signed informed consent and authorization for the use of protected health information were provided by the participants before implementing any protocol‐specific procedures.

## Conflicts of Interest

Stérimar is a registered trademark of Sofibel S.A.S, a subsidiary of Church & Dwight Ltd. C.P. is an R&D Manager at Church & Dwight Co. Inc. W.H. is a Statistician at Church & Dwight Co. Inc. F.D. is a Junior R&D project leader at Church & Dwight Co. Inc. L.F. is a Global Clinical Associate at Church & Dwight Co. O.C. is a Senior Clinical Manager at Church & Dwight Co. Inc. Y.G.B.S. and N.D. have no conflicts of interest to declare.

## Data Availability

Data available on request from the authors.
